# An overview of Indian research in depression

**DOI:** 10.4103/0019-5545.69231

**Published:** 2010-01

**Authors:** Sandeep Grover, Alakananda Dutt, Ajit Avasthi

**Affiliations:** Department of Psychiatry, Postgraduate Institute of Medical Education and Research, Chandigarh, India

**Keywords:** Depression, clinical features, India

## Abstract

Depression as a disorder has always been a focus of attention of researchers in India. Over the last 50-60 years, large number of studies has been published from India addressing various aspects of this commonly prevalent disorder. The various aspects studied included epidemiology, demographic and psychosocial risk factor, neurobiology, symptomatology, comorbidity, assessment and diagnosis, impact of depression, treatment related issues and prevention of depression in addition to the efficacy and tolerability of various antidepressants. Here, we review data on various aspects of depression, originating from India.

## INTRODUCTION

Depression is a disorder of major public health importance, in terms of its prevalence and the suffering, dysfunction, morbidity, and economic burden. Depression is more common in women than men. The report on Global Burden of Disease estimates the point prevalence of unipolar depressive episodes to be 1.9% for men and 3.2% for women, and the one-year prevalence has been estimated to be 5.8% for men and 9.5% for women. It is estimated that by the year 2020 if current trends for demographic and epidemiological transition continue, the burden of depression will increase to 5.7% of the total burden of disease and it would be the second leading cause of disability-adjusted life years (DALYs), second only to ischemic heart disease.[1] In view of the morbidity, depression as a disorder has always been a focus of attention of researchers in India. Various authors have tried to study its prevalence, nosological issues, psychosocial risk factors including life events, sympto matology in the cultural context, comorbidity, psychoneurobiology, treatment, outcome, prevention, disability and burden. Some of the studies have also tried to address various issues in children and elderly.

This review focuses on research done on various depressive disorders in India. For this, a thorough internet search was done using key words like depression, life events, prevalence, classification, cultural issues, outcome, prevention, disability and burden etc in various combinations. The various search engines like Pubmed, Google Scholar, Sciencedirect, Search Medica, Scopus, And Medknow etc were used. In addition thorough search of all the issues of Indian Journal of Psychiatry available online was done. Hand search of some of the missing issues was also attempted and this yielded a few more articles. Review articles which were felt to be not reflecting the Indian scenario to a large extent or not covering the available Indian data were excluded. Treatment issues (antidepressants) are reviewed separately by us in this compilation of annotations to be published. Data from animal studies and originating in the form of case reports and small case series, until felt necessary haven’t been included. The available data has been organized under the headings of epidemiology, demographic and psychosocial risk factors, neurobiology, symptomatology, comorbidity, assessment and diagnosis, impact of depression, treatment related issues and prevention of depression.

### Epidemiology

Many studies have estimated the prevalence of depression in community samples and the prevalence rates have varied from 1.7 to 74 per thousand population.[2,3] Reddy and Chandrasekhar[2] carried out a metanalysis, which included 13 studies on epidemiology of psychiatric disorders which include 33572 subjects from the community and reported prevalence of depression to be 7.9 to 8.9 per thousand population and the prevalence rates were nearly twice in the urban areas.[2] The findings with regard to prevalence in urban population are in line with the findings of a survey done on the entire adult population of an industrial township, which showed that the prevalence rate for depression to be 19.4 per thousand.[4]

A recent large population-based study from South India, which screened more than 24,000 subjects in Chennai using Patient Health Questionnaire (PHQ)-12 reported overall prevalence of depression to be 15.1% after adjusting for age using the 2001 census data.[5] In another recent study, Nandi *et al*.[3] compared the prevalence of depression in the same catchment area after a period of 20 years (first in 1972 and then in 1992) and reported that the prevalence of depression increased from 49.93 cases per 1000 population to 73.97 cases per 1000 population.[3] Studies done in primary care clinics/center have estimated a prevalence rate of 21-40.45%.[6–9] Studies done in hospitals have shown that 5 to 26.7% of cases attending the psychiatric outpatient clinics have depression.[10–13]

Studies on the elderly population, either in the community, inpatient, outpatient and old age homes have shown that depression is the commonest mental illness in elderly subjects.[14–19] Nandi *et al*.[14] studied psychiatric morbidity of the elderly population of a rural community in West Bengal. In a sample of 183 subjects (male 85, female 98) they found 60% of the population to be mentally ill with higher morbidity in women compared to men (77.6% and 42.4% respectively). There was significantly more morbidity in population in the age group 70-74 and 80+ as compared to normal population. The total mental morbidity rate was as high as 612/1000 population. Depression was the commonest illness of old age in this sample, the rate being 522/1000 population (101 cases out of 112 were diagnosed as cases of depression). Women had a higher rate of depression-704/1000 population. Another significant finding was the high rate of morbidity amongst the widowed persons.

An epidemiological study from rural Uttar Pradesh showed that psychiatric morbidity in the geriatric group (43.32%) was higher than in the nongeriatric group (4.66%) and most common psychiatric morbidity was neurotic depression, followed by manic-depressive psychosis depression, and anxiety state. Psychiatric morbidity was more prevalent in those who were socially, economically, and educationally disadvantaged.[15] Recent community-based studies have reported a prevalence rate of 21.7% to 45.9%.[20,21]

Chhabra and Kar[16] studied the profile of psychiatric disorders in elderly psychiatric inpatients and reported that mood disorders were the most common diagnosis (46.5%). Older studies from Gero-psychiatric clinics reported a prevalence of depression ranging from 13 to 22.2%.[22,23] A recent outpatient study, which evaluated psychiatric morbidity in 100 randomly selected elderly subjects attending geriatric clinic, found that 29% patients suffered from psychiatric illness of which depressive disorders were the most common.[17] Another study also reported depression to be the most common psychiatric diagnosis among the 1586 elderly subjects (age ≥60 years), who attended the Geriatric Clinic of the All India Institute of Medical Sciences, New Delhi.[24] In a study of old age home population, Guha and Valdiya[18] reported that major depressive disorder (13.4%) was the most common psychiatric diagnosis in this population.

With regard to epidemiology of depression in children and adolescents in a community sample from south India, Srinath **et al**.[25] reported a prevalence of 0.1% in the 4-16 year age group and no child in the age group 0-3 was diagnosed to have depression. Another study from north India reported an annual incidence rate of 1.61/1000 children in a community based study on school children.[26] Clinic-based studies have reported a prevalence rate of 1.2 to 9.2% for the affective dis orders, amongst which unipolar depression was the commonest category in most of the studies.[27–32] However, in a recent study evaluating the trend of various diagnoses in clinic population, Malhotra **et al**.[33] reported increase in prevalence of affective disorders from 2% to 13.49% in children (0-14 years) attending the psychiatric outpatient clinics. Studies done in women during and after pregnancy have reported incidence of post-natal depression to be 11%.[34]

### Demographic and psychosocial risk factors for depression

In terms of sociodemographic variables studies have shown that depression is more common in women,[4,5,35–37] younger subjects,[13] in subjects from poor economic background[5,36,38] and subjects with poor nutritional status,[38] Muslims,[35] those who are divorced or widowed,[5] those residing in nuclear families[39] and urban areas.[2] Studies which have evaluated the subjects with late onset or old age depression (first episode of depression at or after the age of 50) have also shown that depression is more common in low social class, widowed state,[14,37,40] unemployed condition, low educational level, in subjects living in nuclear family or in those living alone.[15,21,37,40] With regard to gender most of the studies have reported that it is more common in elderly females,[14,37,40] however, some clinic-based studies suggest that it is more common in elderly males.[41] It is also seen that prevalence of depression increases with increasing age in elderly.[20]

Studies have shown that compared to healthy controls and subjects with schizophrenia, depressed patients have significantly greater number of life events prior (6-12 months) to the onset of their illness.[42–46] In terms of type of life events, it is seen that depressed patients experience significantly higher proportion of life events related to death of a family member, personal health related events, bereavement, interpersonal and social events[34,42,44,47] and lower number of life events in the form of illness of family members compared to patients with schizophrenia.[44]

It is also seen that compared to patients with mild depression patients with moderate and severe depression tend to use avoidance as a coping strategies more frequently for the stressful life events, suggesting that it may be a maladaptive way to cope with the situation, which is responsible for development of depression.[43] Studies have also reported that parental loss before the age of 18 years, parental disharmony and eldest birth order tend to be more common in subjects with depression.[48]

Studies in elderly also suggest that life events, especially financial problems and death in the family are as important a precipitating event for depression as they are in young adult.[20,40,49] It is also seen that stressful life events were specifically more in the elderly females and those with lower per capita income.[50]

With respect to life events in children and adolescents, Patel *et al*. found that depressed adolescent girls report life events in the form of death of a family member, change in residence, failure in examination, end of a relationship and serious illness.[51] Other risk factors identified to be associated with depression in children include stress at school and family as well as family history of mental illness.[52] However, one of the older studies failed to find a link between childhood bereavement and depression.[53]

Women as a group have also received considerable attention with regard to risk factors for development of depressive disorders. In an incidence study of common mental disorders, Patel *et al*.[54] reported that poverty (low income and having difficulty in making ends meet), being married as compared with being single, use of tobacco, experiencing abnormal vaginal discharge and reporting a chronic physical illness were associated with risk of developing a common mental disorder.[54] Studies have also reported that economic and interpersonal relationship difficulties, partner violence, sexual coercion by the partner as the common causal factors related to development of depression in general and depression during antenatal and postnatal period.[34,55–58] It has been shown that gender of the newborn child is an important determinant of postnatal depression.[34,56,57]

Among the psychological factors, attribution style was proposed to predispose individuals to depression and maintain depressive symptoms once they develop. A study using the Attribution Style Questionnaire[59] showed that depressed patients have a specific attribution style for their failures and successes in comparison to patients with schizophrenia and medical disorders. According to this study, patients with depression made more internal, stable and global attributions for bad events when compared to other disorders.[60] A study evaluating the cognitive model of depression as given by Beck failed to find support for the causal role of cognitive errors in relapse of depressed subjects as a significant proportion of patients were free from cognitive distortions following remission. However, it was also observed that those who had persistent cognitive distortions during remission ran the risk of early relapse.[61] It has also been seen that patients with neurosis, including depression, have poor social interactions and reports of more interactions of unpleasant type and less of pleasant type of social interactions as compared with healthy controls.[62]

With regard to personality factors, a study showed that higher scores on the hardiness, a personality trait, correlates with lower scores on the depression scale suggesting that presence of hardiness doesn’t allow depressive feelings to become more severe.[63]

### Studies on Neurobiology

Compared to schizophrenia, there is relatively less research on the neurobiology of depression from India.

#### Neurochemicals

Studies have evaluated the levels of catecholamine metabolites in cerebrospinal fluid, urine and blood. Studies have showed that urinary 5-hydroxyindoleacetic acid (5HIAA), a metabolic end product of serotonin was significantly higher in depressed patients as compared to controls and these values decreased following successful treatment.[64,65] However, one study didn’t find any difference in the CSF 5HIAA levels between subjects with depression and healthy controls.[66] Studies have also shown significant positive correlation between the severity of depression and the patient’s urinary 5HIAA values[64,65] and negative correlation between the suicidal ideations and the patient’s urinary and cerebrospinal fluid (CSF) 5HIAA and homovanillic acid (HVA) values.[65] It has also been found that compared to controls, platelet serotonin (5HT) uptake is significantly lower in patients with depression, which improves temporarily with Electroconvulsive Therapy (ECT).[67] Another study by the same authors showed that treatment with Imipramine resulted in significant decrease in platelet 5HT uptake while ECT led to a significant increase in uptake.[68] With regard to dopamine metabolites, it has been seen that HVA levels are low in depressed subjects compared to controls and this correlates with platelet monoamine oxidase (MAO) activity, erythrocyte adenosine deaminase activity and 5-HIAA levels.

Further it was seen that compared to healthy controls HVA/5-HIAA ratio was also lower in subjects with depression.[66] Studies have also shown that both ECT and imipramine cause significant reduction in platelet MAO activity which normalizes during the post-treatment phase.[69] It has been demonstrated that there is significantly low serum dopamine-β-hydroxylase activity in patients with psychotic major depressive disorder compared to healthy controls. However, dopamine-β-hydroxylase activity did not differ between healthy controls, acute schizophrenia patients, subjects with non-psychotic depression and mania.[70] Srinivasan *et al*.[71] also showed that during the acute phase of depression there is low urinary 5-vinyl mandelic acid (VMA), which decrease further during treatment with imipramine. The authors also observed similar finding with respect to effect of imipramine in normal subjects. The levels also normalize in post treatment phase in depressed subjects and after stoppage of treatment in normal subjects.

#### Electrophysiology

Many studies have evaluated the pretreatment P300 amplitude and latency in depressed subjects and compared the same with healthy controls. All studies have consistently shown that P300 amplitude is smaller in depressed subjects and this normalizes with recovery[72–74] suggesting that it may be a state marker of depression. However, the findings with regard to pretreatment P300 latency are inconsistent.[72–74] Studies have also shown that P300 latency has a significant positive correlation with age of the patient and severity of depression while P300 amplitude have a significant negative correlation with age[73] and severity of depression.[72] A study showed that P300 amplitude is lower in depressed subjects, but there is no difference between subjects with depression and dysthymia,[74] however, the pretreatment P300 amplitude does not predict the treatment response.[75]

Studies have also shown that there is no difference between subjects of melancholic depression and control group in the latency of the middle components of evoked potential.[76] Studies done using Bereitschafts potential (BP) a measure of frontal lobe functions have also shown that the amplitude and frequency are lower in subjects with melancholic depression compared to the healthy controls.[76]

#### Endocrinology

As a marker for melancholia, studies have shown that dexamethasone suppression test (DST) has low sensitivity but high specificity.[77–79] Studies have also shown that, compared to DST suppressors, DST non-suppressors are significantly more depressed, attempt suicide more frequently, have higher rates of past and family history of depression, more frequently require electroconvulsive therapy and show better response to treatment.[79] A similar finding with respect to response to treatment has also been reported in another study.[80] Another study on subjects with post-stroke depression showed that DST response parallels the clinical course and response to treatment.[81]

A study of absolute eosinophil counts as an indirect measure of adrenocortical function showed that initial pretreatment level of eosinophils was higher than normal in depressed subjects, which showed a variable response to treatment indicating towards a varying degree of diminished activity of adrenal cortex.[82] Studies have also shown that plasma cortisol levels are higher in depressed subjects compared to healthy subjects and the increase in levels do not correlate with severity of depression.[83] Studies have also demonstrated that abnormal plasma cortisol level can differentiate patients with depression from patients with schizophrenia and healthy controls with a confidence level of 60%-93.3%. Urinary free cortisol after dexamethasone suppression test was considered as the best indicator of differentiating depressed patients from healthy subjects and patients with schizophrenia.[84]

Adrenocorticotrophic Hormone (ACTH) measurements, both at baseline and post-DST in patients of major depression, have been found to be significantly high at baseline and post DST as compared to healthy controls.[85] A study evaluated the role of dexamethasone in treatment based on the hypothesis that dexamethasone would hasten recovery, but did not find any advantage in favor of dexamethasone compared to placebo.[86]

Two studies have found that significantly higher number of patients with unipolar depression have subnormal T3 and T4 levels and a corresponding increase in thyroid stimulating hormone (TSH) levels compared with healthy controls.[87,88] In the second study, mildly depressed patients had significantly lower and severely depressed patients had significantly higher levels of TSH suggesting the direct relationship of severity of depression and TSH levels.[88] Another study found that 20.5% subjects of major depressive disorders have hypothyroidism.[89] When drug naive first episode depression patients were evaluated, they showed significantly higher T4 levels, but there was no significant difference in the level of T3 and TSH between depressed and healthy control subjects. However, when depressed subjects with and without psychotic features were compared, it was seen that subjects with psychotic symptoms had significantly higher levels of TSH.[90]

#### Immunology

It has been shown that there is significant increase in total CSF protein levels in depressed subjects compared to subjects with neurological and surgical illnesses. It was seen that levels of immunoglobulin (Ig) M, IgG and IgA are higher in depressed and neurologically ill subjects compared to subjects with surgical illnesses. Further, when the levels of immunoglobulins were compared between depressed and normal subjects, IgG and IgA were significantly higher in depressed subjects. However, the study did not find evidence of viral markers for rubella and cytomegalovirus in CSF of depressed subjects.[91]

#### Extracellular fluid

Verma and Wig[92] showed that the extracellular fluid (ECF) volume of patients with depression tends to be lower than normal controls and normalization of the same correlates with clinical improvement.

#### Melatonin

Studies have shown that urinary melatonin levels can help in distinguishing subjects of endogenous depression from those with neurotic depression. Nocturnal as well as 24 hours urinary melatonin levels are low in subjects with endogenous depression whereas subjects with neurotic depression have higher than normal levels. It was also seen that melatonin levels were related to suicide attempts, diurnal variation and psychomotor retardation.[93]

#### Neurocognitive functioning

Studies from India suggest that definite cognitive impairments are present in the domains of intelligence and memory (Bhatia’s Battery test or the Weschler Adult Performance Intelligence Scale and PGI memory scale) in the depressed state but these don’t persist following recovery.[94–96] It is also reported that subjects with depression perform poorly on the Wisconsin Card Sorting Test (WCST) as compared to controls suggesting cognitive inflexibility and prefrontal dysfunction. Further, more severe illness is associated with greater impairment in the executive functioning on WCST.[97] Other studies which have evaluated various cognitive domains have shown that when patients with depression are asked to discriminate the emotional tone in terms of intensity of facial expression while presented in pairs, it is seen that they are highly evaluative of sadness and less evaluative of happiness, in comparison to the normal.[98]

#### Genetics

A recent study, showed the importance of genetic factors in treatment response. Significantly better response to escitalopram was seen in patients homozygous for long allele of the serotonin transporter gene compared to the patients who were homozygous for short allele or heterozygous for short and long allele.[99] Another study showed that short variants of D7S1875 marker in LEP gene may be a risk factor for depression.[100]

#### Lipids

A recent study evaluated the serum total cholesterol level in depressed subjects and showed that there is significant elevation of serum total cholesterol in depressed patients compared with normal controls and this persists even after controlling for the confounders.[101] Another study suggested that measurement of serum cholesterol levels may actually indicate towards hypothyroidism in depressed subjects.[89]

### Symptomatology

Many studies have reported the symptom profile of subjects with depressive disorders.[5,13,36,39,52,102–118] The findings of symptomatology in general can be understood with respect to somatic symptoms, guilt and other depressive ideations, suicidal behavior, phenomenology of delusions and sleep architecture. Studies have also compared the symptomatology across different regions of the country. Studies have also attempted to distinguish the phenomenology in depression from negative symptoms of schizophrenia and the phenomenology in dysthymia.

One common theme with regard to symptomatology of depression, which has been reported by most of the researchers is high prevalence of somatic symptoms and some studies report that somatic symptoms are the most common manifestation of depression in India.[5,36,104–106,110,111,119,113,114,122] Erna Hoch[120] also reported that many Indian subjects with depression have hypochondriacal ideas considering body and its functioning. Studies have also shown pain as a depressive equivalent symptom.[42,1] Studies which have compared Indian subjects with depressed subjects from the West have also reported that somatic symptoms are more common in Indian subjects.[104] On the other hand studies on prevalence of functional somatic complaints in patients attending the psychiatric outpatient have also reported that most of these cases are diagnosed as depression.[122,124] It has also been shown that depressed subjects have greater difficulty in identifying bodily sensations and feelings as well as in expressing feelings.[125]

However, some of the studies evaluating depressive symptoms using the standardized instrument have reported that other symptoms are also present quite frequently in depressed subjects. One study which assessed 100 subjects with depression on HDRS reported that depressed mood and difficulties in work are present in all cases. Other symptoms reported in more than 50% of subjects included late insomnia, somatic anxiety, initial insomnia, psychic anxiety, suicidal ideations, retardation, loss of insight, middle insomnia, genital symptoms, hypochondriasis, gastrointestinal symptoms, agitation somatic symptoms in general and diurnal variation. Depersonalization, paranoid and obsessional symptoms were reported very infrequently. Guilt was present in about half of the subjects.[106] Gutpa *et al*.[103] also studied the symptomatology of depression from north India and compared it with findings from south India. Significantly higher number of subjects from north India reported joylessness, disruption in social functioning, lack of self confidence, early morning awakening, lack of appetite, feeling of pressure, other psychological symptoms, psychomotor restlessness, mood worsening in the morning, subjective experience of memory loss, retardation and guilt feeling; significantly higher number of subjects from south India reported hypochondriasis. There was no difference in other symptoms.

Another study from north India evaluated the symptomatology of depression and reported sadness, lack of interest, disturbed sleep, hypochondriasis, poor concentration, agitation, suicidal thoughts, and appetite change as the commonly occurring symptoms. Guilt was reported in about 40% of cases. The authors also compared their findings with studies from other parts of the country and the West and reported that lack of interest was more common in subjects from north India while reduced self-confidence, delusions and suicidal thoughts were seen more often in the south Indian sample.[47] Studies from Mumbai less frequently reported hypochondriasis, guilt, weight change, reduced interest and more frequently reported constipation.[47]

Earlier a few dynamically oriented psychiatrists envisaged guilt as the core symptom of depression. It is also suggested that guilt is less commonly seen in eastern population compared to the west. Studies done in India suggest that it is present in 5.3-67.5% of subjects.[36,47,106,126–129] Bhattacharyya and Vyas[130] also reported lesser frequency of guilt feeling in Indian subjects compared to Australian subjects. Venkoba Rao[127] on the basis of karma theory hypothesized that guilt may not be integral part of depression in Indian subjects and is actually a consequence of depression.[127] Sethi *et al*.[131] also reported that there is no relationship of guilt with severity of depression[131] and Trivedi *et al*.[132] reported higher level of guilt in depressed subjects as compared to neurotic subjects.[132]

Studies have also evaluated suicidal thinking in depressed subjects. Venkoba Rao and Nammalvar[107] reported that about two-third of depressed subjects have suicidal behavior and on the basis of the content they classified suicidal ideation into four broad categories, viz., ideas to kill oneself, a mere wish to die, a wish to be killed and a fourth unclassifiable category. In a recent study on the relationship between anger and suicidality, depressed patients with anger attacks exhibited more suicide-related phenomena in comparison to depressed patients without anger attacks.[133] Studies which have evaluated depressed subjects with suicidal ideation have shown that 16.6% of these subjects make suicidal attempt and a higher risk of suicidal attempt is found in individuals less than 30 years of age, single men, married women and students and higher education.

Attempters scored significantly higher in severity of suicidal ideation, agitation and paranoid symptoms whereas among non-attempters, hypochondriasis and general somatic symptoms were more common.[111] Studies have also shown that depressed subjects who attempt suicide are at higher risk of indulging in further suicidal behavior, compared to those who do not attempt.[134] However, it has also been shown that presence of suicidal behavior does not predict overall poor clinical outcome.[135]

Studies have reported that amongst the delusions in subjects with depression, delusions of persecution occurs most frequently (67.5%) (with persecution involving either the patients themselves or people close to them) followed by delusions of reference. Hypochondriacal, guilt and nihilistic delusions, which are considered classical in depression, are relatively uncommon in Indian subjects.[109]

In terms of sleep architecture, it is reported that subjects with depression have lesser total sleep time, longer sleep latency, frequent awakenings, greater wake-after-sleep onset and offset times, lesser sleep efficiency and tendency to wake up earlier than controls. Subjects with severe depression differ from patients with mild and moderate depression with regards to total sleep time, night-time sleep and sleep efficiency.[136]

Studies which have tried to distinguish depression from negative symptoms have shown that depressed patients score significantly higher on subjective complaints, total score, global ratings on Scale for the Assessment of Negative Symptoms (SANS), while schizophrenia is associated with significantly higher scores on global rating of alogia, poor eye contact, inappropriate affect, and blocking.[115] Another study showed that anhedonia-asociality are seen commonly in both patients with depression and schizophrenia while the global ratings on affective flattening, alogia, avolition and inattention are significantly higher in subjects with schizophrenia.[114]

A study which tried to distinguish the symptomatology of chronic major depression and dysthymia showed that symptomatically dysthymia and chronic major depression are indistinguishable.[137]

Studies have evaluated the symptomatology of depression in elderly depressed subjects too and have reported that the common symptoms in order of frequency were sadness, depressed mood, somatic symptoms and signs, suicidal ideas, lack of energy, anxiety or tension, inability to fall asleep, early awakening, hopelessness, irritability and inability to enjoy.[41] Another study from community sample reported that disturbed sleep pattern is the most common symptom in depressed elderly subjects.[21]

One study which evaluated the symptomatology of depression in children and adolescents reported multiple somatic complaints as the most common presenting complaint in children with depression.[117] Another study, which compared the symptomatology of children and adults, showed that more children than the adults presented with the somatic symptoms and the predominant mood symptom in children was irritability in contrast to sadness in adults.[118] Other commonly reported symptoms of depression across studies include low mood, diminished interest in play and activities, excessive tiredness, low self-esteem, problems with concentration, behavior symptoms like anger and aggression, decreased interest in school and recent deterioration in school performance, death wish and suicidal behavior.[52,117]

In terms of symptomatology in postpartum depression, infanticidal ideas have been reported in depressed mothers.[34] Studies evaluating subjects with seasonal affective disorder found that atypical vegetative features are not prominent part of the symptomatology in India.[138,139]

### Comorbidity

Many studies done in subjects with depression have shown high level of comorbidity of both psychiatric and physical illnesses, especially in elderly individuals with depression.[140,141] On the other hand studies on the prevalence of psychiatric morbidity in physical disorders have shown that depression is quite prevalent in these conditions.

The commonly reported physical illnesses in subjects with depression include those involving the musculoskeletal, cardiovascular and ophthalmological systems and the commonly reported diagnosis in order of frequency were osteoarthritis, hypertension and cataract in one study.[141] The commonly and consistently reported comorbid conditions in children with depression include anxiety and conversion/dissociative disorder.[51,116,117] Other comorbid conditions include, dysthymia, adjustment disorder, conduct disorder and attention deficit hyperactivity disorder.[52,117,118]

Studies done in subjects with intentional self harm,[142] subjects with serious suicide attempts,[143] divorce seeking couples,[144] earth quake victims,[145] women after childbirth,[56] obsessive compulsive disorder,[146] alcohol dependent subjects,[147] dementia,[148] Dhat syndrome,[149,150] medical in-patients,[151–153] epilepsy,[154] neurological disorders,[155] end stage renal disease,[156,157] cancer,[158] chronic and disfiguring skin disorders,[159] age related macular degeneration,[160] HIV-infected heterosexuals,[161] industrial population[162] have shown that depression is the most common or one of the most common psychiatric diagnosis in such patient groups and prevalence rate as high as 86.7% has been reported.

### Assessment and Diagnostic issues

In clinical and research work, apart from uniform diagnostic criteria, some means to objectively quantify the presence of particular symptoms and level of their severity is required. For this purpose, a number of rating scales have been devised worldwide, of which clinician rated Hamilton Depression Rating Scale (HDRS),[163] self reporting Beck Depressive Inventory,[164] Montgomery-Asberg Depression rating scale (MADRS) are the most popular ones. Indian researchers have adapted/modified these scales for the Indian population.[165–167] Additionally scales like Amritsar Depressive Inventory (ADI) a self reporting scale has been developed on the basis of symptoms and signs of depression as manifested by Indian patients.[168] Avasthi *et al*.[169] translated the PRIME-MD questionnaire in Hindi and showed that it is useful for screening various psychiatric disorders. Brief Patient Health Questionnaire (PHQ) has also been translated in 11 languages and validated for Indian population.[170] Many studies have also evaluated the psychometric properties of various scales in India.[171–173]

### Impact of Depression

Studies have shown that depressive disorders lead to significant dysfunction,[174] disability[175] and poor quality of life in sufferers[176] and pose a significant burden on the caregivers.[177] The pattern of burden experienced by relatives of patients with affective disorders and schizophrenia have been shown to be similar, being principally felt in the areas of family routine, leisure, interaction and finances. However, the caregivers of subjects with depression experience lesser degree of burden compared to caregivers of schizophrenia and bipolar disorders.[177,178] Another study showed that the burden of dysthymia is similar to neurotic disorders like obsessive compulsive disorder and generalized anxiety disorder.[179] It has also been seen that patients with dysthymia have significant impairment on measures of quality of life, disability, social support and marital adjustment compared to normal/medically ill controls. The study also showed that duration of illness and severity of depression are the most important correlates of impaired quality of life and disability.[180] A study assessing the relationship of stigma to both depression and somatization in psychiatric patients of south India showed that although both depressive and somatic symptoms were distressing, perceived stigma was more for depressive symptoms. Depressive symptoms were perceived as socially disadvantageous as compared to somatization symptoms.[167]

### Course and Outcome

There are very few studies which have evaluated the course and outcome of depression. Venkoba Rao and Nammalvar[181] followed up 109 out of 122 cases of endogenous depression 3-13 years after their index diagnosis. No recurrence occurred in 28 cases. Forty-two cases turned out to be bipolar and 21 remained unipolar. Manic episodes outnumbered the depressive ones. The change of polarity from depression to mania occurred within three years after the initial depression, though in others the shift occurred between 3 to 12 years. The number of episodes of depression before the onset of mania varied from one to three. While the onset of depression before the age of 40 years predisposed to recurrences, there was risk of chronicity in those patients who developed the illness after 40 years. Gada[182] studied 92 out of 100 cases of major depressive disorder five to 10 years after the index diagnosis and showed that 36.6% cases had no recurrence of episodes and 63.4% had recurrence. Of the total subjects, 37.8% were diagnosed as bipolar disorders and 25.6% were diagnosed as recurrent depressive disorder.

The change of diagnosis from major depressive disorder to bipolar disorder occurred within three years after the initial depression in 77% of cases. In another study Brown *et al*.[183] reported that in their cohort all subjects experienced full recovery within the one-year period. At one-year follow-up, 71% of depressive patients demonstrated no symptoms or social impairment.[183] The mean duration of depressive episode was 14.2 weeks and the rate of relapse was 18%. The authors concluded that overall outcome was considerably more favorable than in comparable studies of affective disorders in developed settings. All these findings suggest that depressive disorders have high chance of recurrence and many of the subjects initially diagnosed as cases of unipolar depression are later diagnosed as bipolar disorder. In another prospective study of subjects with seasonal affective disorder (SAD), Avasthi *et al*.[184] reported that after the initial diagnosis of SAD the subjects did not display any variation in mood, behavior, sleep pattern and weight fluctuation over a period of five to seven years.

Study done in elderly patients with depression have reported that complete recovery occurs in 58% of cases and 24% of cases have partial recovery with 18% registering relapses.[41] Sachdev *et al*.[185] studied various factors related to prognosis of depression and found that sleep-disturbances, agitation, depth of depression, age at the time of present onset, age at first onset, history over one year with no symptom-free period, sudden onset of illness, adequate premorbid personality and adequate psychogenesis are of prognostic significance.

### Treatment related issues

One of the important patient characteristic which has been reported to influence treatment adherence is their attitudes and beliefs towards medication. In a recent study, Chakraborty *et al*.[186] reported that most of the patients value the doctor-patient relationship and their partners are also supportive regarding diagnosis and treatment of depression. However, most patients have erroneous beliefs regarding antidepressants per se which in turn influence the drug compliance.

### Prevention

Sethi *et al*.[187] proposed a prevention model for depression focusing on improving social network and educational programs, designed to educate the public with regard to the risks inherent to change of jobs, residence, pattern of living as well as how to protect against them and removal of malnutrition and infections. A recent review suggests that religiosity can be protective against depression.[188]

### Conclusion and future directions

Depression is the most common psychiatric disorder reported in most of the community based studies. It is also reported as one of the most common psychiatric disorder in outpatient clinic population and in subjects seen in various medical and surgical setting. It is also reported to be the most common psychiatric disorder in elderly subjects across various settings. Studies from India have also shown that life events during the period preceding the onset of depression play a major role in depression. Studies on women have also shown the importance of identifying risk factors like interpersonal conflicts, marital disharmony and sexual coercion.

There is need for further study of factors like cost, attitude towards treatment, adherence, compliance and neurobiological correlates. There is also a need to study the course of depressive disorders in India so as to determine the need and duration of continuation treatment. Studies should also evaluate the cost-effective models of treatment which can be easily used in the primary care setting to effectively treat depression.
